# Elevated Serum Leptin Levels as a Predictive Marker for Polycystic Ovary Syndrome

**DOI:** 10.3389/fendo.2022.845165

**Published:** 2022-03-09

**Authors:** Yuanyuan Peng, Hongyue Yang, Jiahui Song, Di Feng, Zhijing Na, Hongyu Jiang, Yaxin Meng, Bei Shi, Da Li

**Affiliations:** ^1^ Center of Reproductive Medicine, Shengjing Hospital of China Medical University, Shenyang, China; ^2^ Education Center, Clinical Skill Practice, China Medical University, Shenyang, China; ^3^ Medical Basic Experimental Teaching Center, China Medical University, Shenyang, China; ^4^ Key Laboratory of Reproductive Dysfunction Diseases and Fertility Remodeling of Liaoning Province, China Medical University, Shenyang, China

**Keywords:** leptin, polycystic ovary syndrome, insulin resistance, hyperandrogenism, monosaccharide, metabolic disorder, obesity

## Abstract

**Background:**

Leptin may have important implications in polycystic ovary syndrome (PCOS)-related metabolic disorders. However, the changes in serum leptin levels in patients with PCOS and its predictive value for PCOS remain obscure. We intend to analyze the association between leptin and PCOS in this study.

**Materials and Methods:**

The study comprised 89 patients with PCOS and 139 individuals without PCOS. Each group was stratified as either normal- or hyper-fasting serum insulin (FSI), and lean or overweight/obese; and the patients were further categorized as normal- or hyper-androgenic. The validity of leptin toward the diagnosis of PCOS, or leptin combined with total testosterone, dehydroepiandrosterone sulfate (DHEAS), and free testosterone was estimated by receiver operating characteristic (ROC) curves, and correlations between paired variables was estimated by Spearman’s rank correlation coefficient. Associations between the clinical and metabolic variables and PCOS were analyzed *via* logistic regression.

**Results:**

The serum leptin levels of patients with PCOS were significantly higher than that of the control, and especially the PCOS in hyper-FSI, hyperandrogenimic and overweight/obese subgroups. The area under the ROC curve (AUC) of leptin was 74%, with cutoff value, sensitivity, specificity, positive predictive value (PPV) and negative predictive value (NPV) 11.58 ng/mL, 77.5%, 62.6%, 57.0%, and 81.3%, respectively. Combined leptin and anti-Müllerian hormone (AMH) had the highest AUC (92.3%), excellent sensitivity (93.3%), moderate specificity (78.3%), PPV (73.5%) and NPV (94.8%). Serum leptin levels of the patients were correlated with the FSI, fasting plasma glucose (FPG), homeostasis model assessment of insulin resistance (HOMA-IR), body mass index (BMI), and total testosterone levels. Elevated serum leptin was associated with a high risk of PCOS [*P* = 0.015; OR (95% CI) 1.128 (1.024–1.244)].

**Conclusion:**

Substantially elevated serum leptin is significantly associated with PCOS. These findings warrant further investigations into the function of leptin in the pathogenesis of PCOS.

## Introduction

Polycystic ovary syndrome (PCOS) is a heterogeneous disease involving metabolic and endocrine dysregulation with a worldwide prevalence of 5 to 20% (1). Various symptoms affecting women of reproductive age include hyperandrogenism, insulin resistance, obesity and ovulation dysfunction (2, 3). Monosaccharides are the simplest of the carbohydrates and the major energy source for metabolism (4). Our previous study (5) used RNA sequencing to show that the biosynthesis of monosaccharides is a novel pathway marker for differentiating PCOS. Furthermore, we found (6) that patients with PCOS were characterized with elevated serum levels of fructose which is an important reducing monosaccharide. Vasselli et al. (7) proposed that long-term intake of fructose can induce leptin resistance by reducing phosphorylation.

Leptin is a fat cell-derived hormone that promotes a shift from carbohydrate to fat oxidation and regulates glucose homeostasis (8). However, abnormal levels of leptin can lead to a variety of endocrine and metabolic dysfunctions. The importance of leptin in PCOS-related endocrine and metabolic disorders has been gradually recognized. For example, mounting evidence has indicated that circulating leptin levels in obese patients increase over a long period of time, reducing the sensitivity of leptin receptors and increasing leptin resistance (9). Reduced leptin sensitivity can result in the accumulation of excess triglycerides in the adipose tissue, liver, muscle, and pancreas, causing impaired insulin sensitivity and secretion and eventually insulin resistance (10). In addition, high leptin levels may also affect follicular development and fertility (11, 12). Currently, no consistent conclusion and systematic analysis exists with regard to changes in serum leptin levels in PCOS (13, 14). The present study aims to investigate the association between leptin and PCOS and the predictive value of leptin for PCOS.

## Materials and Methods

### Patients and Blood Samples

Overall, 228 Chinese women of matching age were enrolled in this study at Shengjing Hospital of China Medical University, comprising 89 patients with PCOS and 139 healthy women (control group). Blood samples were collected in the morning, after overnight fasting, and between the third and fifth days of spontaneous menstrual bleeding. The diagnosis of PCOS was made as per the Rotterdam standards (two out of three):1. oligo‐ and/or anovulation 2. clinical and/or biochemical signs of hyperandrogenism 3. polycystic ovaries (15). The control group consisted of women with tubal infertility or male-partner infertility. Patients who were pregnant or lactating, on hormone medication and medications (corticosteroids, oral contraceptives, aspirin, insulin-sensitizing drugs, nicotinic acid, anti-androgens, statins) within 6 months, and with endocrine diseases such as androgen-secreting tumor, diabetes, Cushing’s syndrome, congenital adrenal hyperplasia, and hyperprolactinemia, were excluded. The full list of exclusion criteria is the same as that specified in our previous articles (5, 6).

To measure the leptin levels in patients with different types of PCOS, the control group and PCOS patients in our cohort were subdivided into the normal-fasting serum insulin (FSI) and hyper-FSI groups. This subdivision was chosen because it has been proposed in recent literature that the FSI level can be regarded as a precise alternative predictor of insulin resistance in PCOS patients, with an accuracy similar to fasting plasma glucose (FPG)/FSI and the homeostatic model assessment of insulin resistance (HOMA-IR) values (16). The reference ranges of the FSI levels were determined at Shengjing Hospital of China Medical University. The diagnosis of biochemical hyper-FSI was made when the FSI was ≥ 11.8 mIU/L. The characteristics of each FSI subgroup are shown in **Table 1**.

**Table 1:** Description of the study participants subdivided by different levels of FSI.

	Normal-FSI			Hyper-FSI		
	Control (n = 80)	PCOS (n = 32)	*P*-value	Control (n = 59)	PCOS (n = 57)	*P*-value
Age (year)	31.00 (29.00-33.00)	31.50 (30.00-32.75)	NS	31.00 (29.00-34.00)	32.00 (30.00-34.00)	NS
BMI (kg/m^2^)	22.90 (21.53-24.30)	23.55 (22.00-25.68)	NS	25.60 (24.00-28.00)	27.20 (24.65-29.02)	NS
Leptin (ng/mL)	7.98 (4.95-11.45)	12.27 (9.43-16.83)	*P* < 0.001	13.29 (9.01-16.86)	15.66 (14.02-19.52)	*P* < 0.05
FPG (mM)	5.08 (4.90-5.30)	5.04 (4.70-5.40)	NS	5.45 (5.13-5.73)	5.22 (4.99-5.66)	NS
FSI (mIU/L)	9.25 (6.95-10.28)	8.95 (7.43-10.18)	NS	15.50 (14.00-19.60)	19.00 (13.60-23.40)	NS
HOMA-IR	2.10 (1.54-2.37)	1.95 (1.56-2.42)	NS	3.79 (3.20-4.92)	4.36 (3.40-5.83)	NS
Free testosterone (nmol/L)	0.021 (0.016-0.027)	0.030 (0.018-0.038)	*P* < 0.05	0.026 (0.019-0.034)	0.031 (0.023-0.042)	*P* < 0.05
DHEAS (nmol/L)	2961 (1965-3695)	4059 (2830-5592)	*P* < 0.05	3307 (2217-4502)	4519 (3039-6375)	*P* < 0.05
Total testosterone (ng/mL)	0.48 (0.35-0.57)	0.63 (0.54-0.73)	*P* < 0.001	0.51 (0.37-0.61)	0.73 (0.52-0.87)	*P* < 0.001
FSH (mIU/L)	7.26 (6.17-8.73)	6.45 (5.73-7.51)	*P* < 0.05	6.93 (5.58-7.64)	6.26 (5.12-7.78)	NS
LH (mIU/L)	3.84 (2.96-5.09)	11.31 (5.80-11.01)	*P* < 0.001	3.65 (2.88-5.39)	9.43 (5.27-13.44)	*P* < 0.001
LDL-C (mM)	2.65 ± 0.70	3.04 ± 0.70	*P* < 0.05	2.71 ± 0.69	3.08 ± 0.76	*P* < 0.05
HDL-C (mM)	1.28 (1.12-1.53)	1.30 (1.08-1.50)	NS	1.13 (0.95-1.33)	1.06 (0.90-1.17)	NS
Prolactin (ng/mL)	10.35 (8.45-13.48)	8.80 (7.59-11.01)	*P* < 0.05	10.40 (7.83-13.47)	9.38 (6.95-12.54)	NS
Progestin (mIU/mL)	0.55 (0.33-0.69)	0.53 (0.36-0.88)	NS	0.53 (0.36-0.81)	0.56 (0.35-1.11)	NS
AMH (ng/mL)	3.08 (1.28-4.54)	9.49 (7.16-12.31)	*P* < 0.001	2.95 (1.50-5.41)	7.10 (4.79-11.12)	*P* < 0.001
Estrogen (pg/mL)	46.00 (35.00-65.25)	52.00 (38.00-69.00)	NS	47.00 (38.50-62.50)	53.00 (40.00-67.00)	NS
Total cholesterol (mM)	4.49 ± 0.78	4.79 ± 0.87	NS	4.39 ± 0.84	4.94 ± 0.83	*P* < 0.05
Triglycerides (mM)	1.28 (1.12-1.53)	1.15 (0.92-1.53)	*P* < 0.05	1.39 (0.91-1.92)	1.90 (1.19-2.48)	*P* < 0.05
TSH (µIU/mL)	1.81 (1.38-2.49)	1.72 (1.20-2.08)	NS	1.89 (1.51-2.72)	1.80 (1.18-2.36)	NS

AMH, anti-Müllerian hormone; BMI, body mass index; DHEAS, dehydroepiandrosterone sulfate; FSH, follicle-stimulating hormone; FSI, fasting serum insulin; FPG, fasting plasma glucose; HDL-C, high-density lipoprotein cholesterol; HOMA-IR, homeostasis model assessment of insulin resistance; LDL-C, low-density lipoprotein cholesterol; LH, luteinizing hormone; TSH, thyroid-stimulating hormone; NS, not significant. Mean ± standard deviation or median (interquartile range) are shown. The Mann-Whitney U test was used for non-normally distributed data, and Student’s t test was used for normally distributed data.

To illustrate the relationship between leptin and androgens, participants in the PCOS group were further divided into the normal-androgenic and hyperandrogenic groups according to serum androgen levels. Total testosterone > 0.68 ng/mL, free testosterone > 0.040 nmol/L, and/or dehydroepiandrosterone sulfate (DHEAS) > 7025.11 nmol/L were used as the diagnostic criterion for biochemical hyperandrogenism. These parameters represented the 95th percentile of basal serum androgens in 139 control participants without PCOS (17). The characteristics of each androgenic subgroup are shown in **Table 2**.

**Table 2:** Description of the study participants subdivided by different levels of androgens.

	Control (n = 139)	Normal-androgenic PCOS (n = 35)	Hyperandrogenic PCOS (n = 54)	*P* ^a^-value	*P* ^b^-value
Age (year)	31.00 (29.00-33.00)	32.00 (30.00-33.00)	31.50 (30.00-33.25)	NS	NS
BMI (kg/m^2)^	24.00 (22.20-26.30)	24.97 (22.80-27.34)	26.30 (23.73-29.01)	NS	NS
Leptin (ng/mL)	9.71 (6.85-14.45)	14.44 (9.96-15.95)	15.84 (12.30-20.33)	*P* < 0.001	*P* < 0.05
FPG (mM)	5.18 (4.98-5.55)	5.00 (4.82-5.43)	5.33 (5.05-5.54)	*P* < 0.05	*P* < 0.05
FSI (mIU/L)	11.00 (8.50-14.90)	11.50 (8.70-19.10)	15.80 (11.50-22.80)	NS	*P* < 0.05
HOMA-IR	2.54 (1.97-3.58)	2.56 (1.86-4.09)	3.81 (2.75-5.30)	NS	*P* < 0.05
Free testosterone (nmol/L)	0.023 (0.017-0.030)	0.022 (0.017-0.032)	0.038 (0.026-0.044)	NS	*P* < 0.001
DHEAS (nmol/L)	3108 (2040-4012)	3283 (2385-4884)	4867 (3157-6512)	*P* < 0.05	*P* < 0.05
Total testosterone (ng/mL)	0.49 (0.36-0.59)	0.53 (0.43-0.62)	0.79 (0.70-0.91)	*P* < 0.05	*P* < 0.001
FSH (mIU/L)	7.08 (5.70-8.27)	6.09 (5.01-7.42)	6.44 (5.64-7.74)	*P* < 0.05	NS
LH (mIU/L)	3.70 (2.92-5.22)	9.07 (5.07-12.98)	11.33 (5.75-15.58)	*P* < 0.001	NS
LDL-C (mM)	2.68 ± 0.70	3.05 ± 0.79	3.08 ± 0.70	*P* < 0.05	NS
HDL-C (mM)	1.22 (1.04-1.41)	1.11 (0.95-1.40)	1.10 (0.96-1.35)	NS	NS
Prolactin(ng/mL)	10.40 (8.24-13.47)	9.54 (7.56-11.57)	9.33 (7.04-12.44)	NS	NS
Progestin (mIU/mL)	0.54 (0.34-0.75)	0.49 (0.34-0.70)	0.66 (0.37-1.01)	NS	NS
AMH (ng/mL)	2.99 (1.43-4.76)	8.63 (5.98-11.77)	8.13 (4.81-11.62)	*P* < 0.001	NS
Estrogen (pg/mL)	47.00 (36.00-65.00)	53.00 (35.75-86.00)	52.50 (41.00-67.00)	NS	NS
Total cholesterol (mM)	4.45 ± 0.80	4.84 ± 0.84	4.92 ± 0.85	*P* < 0.05	NS
Triglycerides (mM)	1.00 (0.71-1.57)	1.53 (0.93-1.93)	1.42 (1.00-2.21)	*P* < 0.05	NS
TSH (µIU/mL)	1.82 (1.46-2.57)	1.83 (1.16-2.33)	1.69 (1.23-2.21)	NS	NS

AMH, anti-Müllerian hormone; BMI, body mass index; DHEAS, dehydroepiandrosterone sulfate; FSH, follicle-stimulating hormone; FSI, fasting serum insulin; FPG, fasting plasma glucose; HDL-C, high-density lipoprotein cholesterol; HOMA-IR, homeostasis model assessment of insulin resistance; LDL-C, low-density lipoprotein cholesterol; LH, luteinizing hormone; TSH, thyroid-stimulating hormone; NS, not significant. The Mann-Whitney U test was used for non-normally distributed data, and Student’s t test was used for normally distributed data. ^a^Comparing the normal-androgenic PCOS patients and controls after post-hoc test. ^b^Comparing normal-androgenic PCOS patients and hyperandrogenic PCOS patients after post-hoc test.

To analyze associations between leptin and obesity in PCOS, the control group and PCOS patients were each analyzed as lean and overweight/obese subgroups. Body mass index (BMI) ≥ 23 kg/m^2^ of female is diagnostic for overweightness/obesity in Asians (18). Characteristics of each subgroup are shown in **Supplementary Table 2**.

### Measurement of Clinical and Metabolic Indicators

The concentration of human leptin in each sample was measured using the Human Leptin ELISA Kit (CSB-E04649h, Cusabio Biotech, Wuhan, China) with a quantitative sandwich-based enzyme immunoassay technique. Each serum sample was diluted 1:5 with the sample diluent before the assay was performed, in accordance with the standard procedure. The detection range was between 0.156 ng/mL–10 ng/mL, and the intra- and inter-assay precision values were less than 8% and 10%, respectively. The sample concentrations were determined by comparison with a standard curve at 450 nm optical density.

The serum concentrations of free testosterone (CSB-E05096h, Cusabio Biotech, Wuhan, China) and DHEAS (CSB-E05105h, Cusabio Biotech, Wuhan, China) were determined using commercial enzyme-linked immunosorbent assay kits, according to the manufacturers’ standard protocols. The intra-assay coefficients of variation for free testosterone and DHEAS were 6.8% and 5.5%, respectively, and the inter-assay coefficients of variation were 10.2% and 8.3%, respectively. The serum FSI levels were measured using the Architect Insulin Reagent Kit (8K41, Abbott Laboratories, IL, USA). FPG and the lipid profiles, including total cholesterol, triglycerides, high- and low-density lipoprotein cholesterol, were detected using enzymatic methods. Total testosterone, luteinizing hormone (LH), anti-Müllerian hormone (AMH), follicle-stimulating hormone (FSH), estradiol, prolactin, thyroid-stimulating hormone, and progestin were measured using chemiluminescence analysis.

### Statistical Analysis

All the data were analyzed using SPSS version 22 (IBM Corp., Armonk, NY). The normality of the continuous variable distribution was measured using the Kolmogorov–Smirnov test, and variables with a normal distribution were represented by means ± standard deviations. Student’s *t* test was applied to ascertain the differences of the means. The Mann–Whitney *U* test was conducted for continuous variables that did not conform to a normal distribution, and the data are expressed as medians (interquartile ranges). Spearman’s rank correlation coefficient was used to determine the correlation intensity between the pairs of variables, and the relationship between the clinical and metabolic variables and PCOS was analyzed using a multiple logistic regression model. The validity of leptin toward the diagnosis of PCOS, or leptin combined with total testosterone, DHEAS, free testosterone and AMH was estimated by receiver operating characteristic (ROC) curves. The area under the ROC curve (AUC) with specificity, sensitivity, negative predictive value (NPV), and positive predictive value (PPV) and 95% confidence interval (CI) were computed in order to analyze the diagnostic efficacy of the various indexes for PCOS. The optimal cutoff value was determined by calculating the Youden index. All the tests were bilateral, and statistical significance was defined as *P* < 0.05.

## Results

### Patients With PCOS Show Higher Serum Leptin Levels Than Controls

The serum leptin levels of the overall PCOS group were significantly higher than that of the control group (**Supplementary Table 1**).

**Figure 1:**
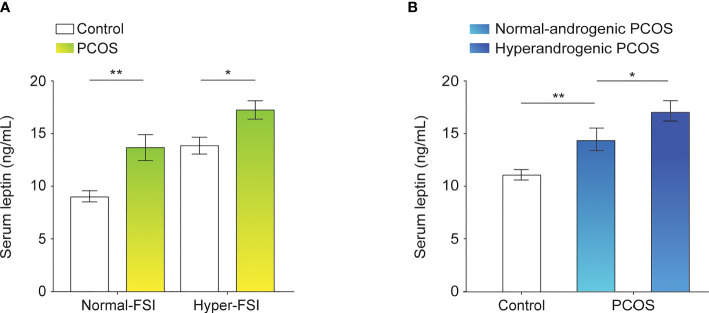
Leptin levels in the serum of control participants and PCOS patients. **(A)** Differences in serum leptin levels between normal-FSI and hyper-FSI control participants and PCOS patients. **(B)** Differences in serum leptin levels between normal-androgenic and hyperandrogenic PCOS patients and control participants. Bar graphs show the mean ± standard error. **P < 0.01, *P < 0.05. PCOS, polycystic ovary syndrome; FSI, fasting serum insulin.

To systematically analyze the association between serum leptin levels and PCOS in the context of FSI, subjects in each of the study groups, PCOS and control, were categorized into normal-FSI or hyper-FSI subgroups. In both the normal-FSI and hyper-FSI subgroups, the serum leptin levels in PCOS patients were markedly higher than those in the control participants. In the hyper-FSI subgroup, the serum leptin levels in the PCOS patients were also significantly higher than those in the normal-FSI subgroup (*P* < 0.001). Moreover, PCOS patients in the hyper-FSI subgroup had the highest leptin levels compared to the other subgroups. The clinical and biochemical characteristics of the subgroups are presented in **Figure 1A** and **Table 1**.

The PCOS group was divided into the normal-androgenic and hyperandrogenic subgroups. As shown in **Figure 1B** and **Table 2**, PCOS patients in both normal-androgenic and hyperandrogenic subgroups had higher serum leptin levels than the control participants. Additionally, PCOS patients in the hyperandrogenic group had higher serum leptin levels than those in the normal-androgenic group.

As shown in **Supplementary Table 2**, control and PCOS groups were divided into the lean and overweight/obese subgroups. Serum leptin levels were significantly increased in the overweight/obese group relative to the lean group in PCOS patients (*P* < 0.001). Moreover, serum leptin levels were significantly higher in PCOS patients than control subjects in the overweight/obese subgroup.

### Prediction of PCOS *via* Serum Leptin Alone or Combined With Various Hormones

**Figure 2:**
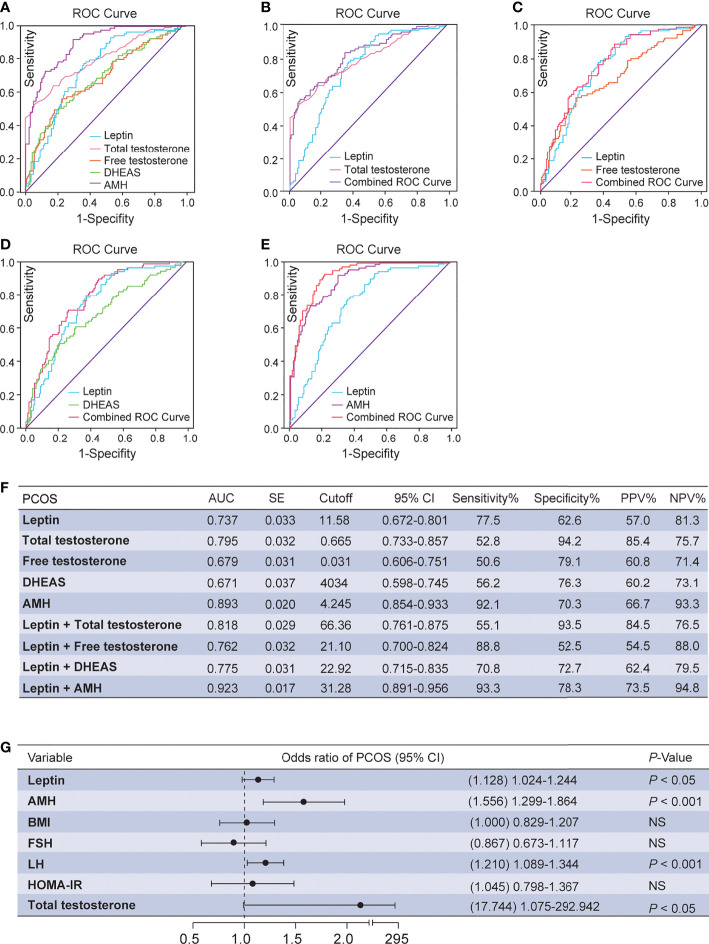
ROC analysis and multivariate logistic regression between serum leptin and androgens. **(A)** Diagnostic potential of leptin, total testosterone, free testosterone, DHEAS and AMH for PCOS estimated using ROC analysis. **(B)** Combined curve of leptin with total testosterone. **(C)** Combined curve of leptin with free testosterone. **(D)** Combined curve of leptin with DHEAS. **(E)** Combined curve of leptin with AMH. **(F)** AUC, SE, cutoff value, 95% CI, Sen%, Spe%, PPV%, and NPV% of ROC curves. **(G)** Multivariate analysis with the following variables in the model: leptin, HOMA-IR, BMI, FSH, LH, total testosterone and AMH. DHEAS, dehydroepiandrosterone sulfate; PCOS, polycystic ovary syndrome; ROC, receiver operating characteristic; AUC, area under the curve; SE, standard error; 95% CI, 95% confidence interval; Sen%, sensitivity %; Spe%, specificity %; PPV, positive predictive value; NPV, negative predictive value; FSI, fasting serum insulin; BMI, body mass index; FSH, follicle-stimulating hormone; LH, luteinizing hormone; AMH, anti-Müllerian hormone; HOMA-IR, homeostasis model assessment of insulin resistance; NS, not significant.

ROC curves were used to estimate the diagnostic performance of leptin, which was then compared and combined with the levels of total testosterone, DHEAS, free testosterone and AMH (**Figures 2A, F**). The AUC of leptin was 73.7%, with a cutoff value of 11.58 ng/mL, and sensitivity and specificity of PCOS prediction of 77.5% and 62.6%, respectively. The PPV and NPV were 57.0% and 81.3%, respectively. However, the combination of leptin and total testosterone resulted in a higher AUC of 81.8%, with poor sensitivity (55.1%) and excellent specificity (93.5%) for the prediction of PCOS (**Figures 2B, F**). The PPV and NPV were 84.5% and 76.5%, respectively. When leptin and free testosterone were combined, the AUC was 76.2%, with excellent diagnostic sensitivity (88.8%) and poor specificity (52.5%) for predicting PCOS (**Figures 2C, F**). The AUC of PCOS was predicted to be 77.5% for the combination of leptin and DHEAS, with an intermediate diagnostic sensitivity (70.8%) and a moderate diagnostic specificity (72.7%) (**Figures 2D, F**). Notably, the combination of leptin and AMH resulted in the highest AUC of 92.3%, with excellent sensitivity (93.3%) and moderate specificity (78.3%) for predicting PCOS. The PPV and NPV were 73.5% and 94.8%, respectively (**Figures 2E, F**). Moreover, the multivariate logistic regression revealed that an elevation of serum leptin level was correlated with a high risk of PCOS (*P* = 0.015; odds ratio, 1.128; 95% CI, 1.024–1.244; **Figure 2G**).

### Relationship of Leptin Levels and Clinical and Metabolic Parameters

Correlation analyses were used between leptin levels and the other parameters in PCOS women (**Supplementary Table 3**). The serum leptin levels exhibited an association with FSI, FPG, HOMA-IR, BMI and total testosterone. There was a moderate and significant correlation between leptin and BMI (r = 0.695) and HOMA-IR (r = 0.415), whereas the correlation was weak between leptin and FSI (r = 0.383), FPG (r = 0.286), and total testosterone (r = 0.308).

## Discussion

This study for the first time systematically evaluated the serum leptin levels of subgroups of a PCOS population. It was found that in patients with PCOS, elevated serum leptin level was associated with hyperandrogenemia and insulin resistance. Thus, leptin may serve as an important biochemical indicator for the diagnosis and treatment of PCOS.

Recent reports shed light on the function of leptin and have identified leptin as having associations with PCOS (19). This study indicated that serum leptin levels were elevated in patients with PCOS compared with the non-PCOS control group, supporting the previous studies. It has been suggested that high leptin in PCOS may interfere with the development of the mature oocyte and ovarian steroidogenesis, contributing to ovulatory dysfunction and infertility (20, 21). Moreover, hyperleptinemia may interact with hyperandrogenemia and chronic low-grade inflammation in PCOS, forming a vicious circle in PCOS (22). Collectively, these findings indicate that leptin might play a role in the occurrence and development of PCOS.

The study indicated a positive association between serum leptin levels and total testosterone levels. There is evidence that rats with dihydrotestosterone-induced PCOS showed a decrease in leptin synthesis and function in adipocytes and enervated leptin activity by increasing the levels of the soluble leptin receptor, a leptin-binding protein, in the hypothalamus. This manifested as obesity and increased circulating leptin levels, accompanied by leptin resistance (23). These observations suggest that androgens may be involved in the pathogenesis of elevated serum leptin levels, and that leptin may further aggravate the PCOS phenotype, as a secondary response to the increase in the androgen levels. High leptin levels can inhibit aromatase activity, which is a key enzyme in the conversion of androgens to estradiol, further aggravating hyperandrogenemia, which affects follicle growth and development, resulting in ovulation dysfunction (22). These findings suggest that the mechanism of leptin involvement in PCOS pathology is related to androgen.

It is well accepted that hyperinsulinemia and insulin resistance are important features in PCOS (24). Risk factors of insulin resistance in PCOS patients include obesity and those related to androgen (25). In addition, the serum leptin concentration has shown a strong positive association with insulin resistance (26). Consistent with these studies, we found that serum leptin levels had a significant correlation with FSI and HOMA-IR levels. Moreover, a growing body of data has suggested that elevated serum leptin levels may play a role in the increase in insulin resistance in obesity (27). Additionally, a partial reduction in plasma leptin levels can restore hypothalamic leptin sensitivity and effectively decrease weight gain as well as improve insulin sensitivity in obesity (28). These findings indicate that the mechanism by which leptin is involved in the development of PCOS involves insulin sensitivity. Thus, it is evident that serum leptin levels may be a useful marker of insulin resistance.

The major strengths of this study: First, this is a systematic assessment of the association between leptin levels and multiple subtypes of PCOS in a study population and provides needed data regarding serum leptin and PCOS subtypes. Secondly, this study includes an assessment of the diagnostic performance of serum leptin levels in PCOS, alone or combined with androgen. This is novel data among published reports. Finally, this study can provide guidance for the diagnosis and treatment of different subtypes of PCOS. This study also has some limitations including the small size of the studied samples. Further studies are needed with large sample size.

This study highlights elevated serum leptin levels in PCOS, and associations between serum leptin and PCOS-related hyperandrogenemia and insulin resistance. Detecting leptin levels may have an important clinical application value for predicting PCOS and its long-term complications. However, it is not yet clear how leptin may interact with androgens and insulin in the pathogenesis of PCOS, which is a topic worth further investigation.

## Data Availability Statement

The raw data supporting the conclusions of this article will be made available by the authors, without undue reservation.

## Ethics Statement

The studies involving human participants were reviewed and approved by Institutional Review Board at China Medical University. The patients/participants provided their written informed consent to participate in this study.

## Author Contributions

DL and BS conceived and designed the study. DL, BS, YP, HY, JS, DF, ZN, HJ, and YM performed data acquisition and interpretation. DL, BS, YP, and HY wrote the paper. All authors contributed to the article and approved the submitted version.

## Funding

This work was supported by the National Natural Science Foundation of China (No. 82071607 and 32100691); LiaoNing Revitalization Talents Program (No. XLYC1907071); Fok Ying Tung Education Foundation (No. 151039); Outstanding Scientific Fund of Shengjing Hospital (No. 202003).

## Conflict of Interest

The authors declare that the research was conducted in the absence of any commercial or financial relationships that could be construed as a potential conflict of interest.

## Publisher’s Note

All claims expressed in this article are solely those of the authors and do not necessarily represent those of their affiliated organizations, or those of the publisher, the editors and the reviewers. Any product that may be evaluated in this article, or claim that may be made by its manufacturer, is not guaranteed or endorsed by the publisher.
