# Supplementary material for: Elevated Serum Leptin Levels as a Predictive Marker for Polycystic Ovary Syndrome
Source: Front Endocrinol (Lausanne). 2022 Mar 9;13:845165. doi: 10.3389/fendo.2022.845165 (PMC8959426; doi:10.3389/fendo.2022.845165)
Supplement: Supplementary file 1 [file Table_1.docx]

**Supplementary Table 1.** Comparison of clinical and metabolic profile of patients with polycystic ovary syndrome (PCOS) and controls.

|  | Control (n = 139) | PCOS (n = 89) | *P*-value |
| --- | --- | --- | --- |
| Age | 31.00 (29.00-33.00) | 32.00 (30.00-33.00) | NS |
| BMI (kg/m^2^) | 24.00 (22.20-26.30) | 25.70 (23.42-28.30) | *P* < 0.01 |
| Leptin (ng/mL) | 9.71 (6.85-14.45) | 15.20 (11.68-18.23) | *P* < 0.001 |
| FPG (mM) | 5.18 (4.98-5.55) | 5.18 (4.88-5.48) | NS |
| FSI (mIU/L) | 11.00 (8.50-14.90) | 13.40 (9.95-21.05) | *P* < 0.01 |
| HOMA-IR | 2.54 (1.97-3.58) | 3.35 (2.33-4.86) | *P* < 0.01 |
| Free testosterone (nmol/L) | 0.023 (0.017-0.030) | 0.031 (0.022-0.040) | *P* < 0.001 |
| DHEAS (nmol/L) | 3108 (2040-4012) | 4318 (2973-5895) | *P* < 0.001 |
| Total testosterone (ng/mL) | 0.49 (0.36-0.59) | 0.68 (0.53-0.84) | *P* < 0.001 |
| FSH (mIU/L) | 7.08 (5.70-8.27) | 6.36 (5.42-7.65) | *P* < 0.05 |
| LH (mIU/L) | 3.70 (2.92-5.22) | 10.48 (5.66-14.83) | *P* < 0.001 |
| LDL-C (mM) | 2.68 ± 0.70 | 3.07 ± 0.73 | *P* < 0.001 |
| HDL-C (mM) | 1.22 (1.04-1.41) | 1.11 (0.96-1.36) | *P* < 0.05 |
| Prolactin (ng/mL) | 10.40 (8.24-13.47) | 9.33 (7.27-12.25) | *P* < 0.05 |
| Progestin (mIU/mL) | 0.54 (0.34-0.75) | 0.55 (0.36-0.90) | NS |
| AMH (ng/mL) | 2.99 (1.43-4.76) | 8.20 (5.18-11.64) | *P* < 0.001 |
| Estrogen (pg/mL) | 47.00 (36.00-65.00) | 53.00 (39.50-68.25) | NS |
| Total cholesterol (mM) | 4.45 ± 0.80 | 4.89 ± 0.85 | *P* < 0.001 |
| Triglycerides (mM) | 1.00 (0.71-1.57) | 1.48 (1.00-2.03) | *P* < 0.001 |
| TSH (µIU/mL) | 1.82 (1.46-2.57) | 1.72 (1.19-2.24) | *P* < 0.05 |

**Abbreviations:** AMH, anti-Müllerian hormone; BMI, body mass index; DHEAS, dehydroepiandrosterone sulfate; FSH, follicle-stimulating hormone; FSI, fasting serum insulin; FPG, fasting plasma glucose; HDL-C, high-density lipoprotein cholesterol; HOMA-IR, homeostasis model assessment of insulin resistance; LDL-C, low-density lipoprotein cholesterol; LH, luteinizing hormone; TSH, thyroid-stimulating hormone; NS, not significant. Mean ± standard deviation or median (interquartile range) are shown. The Mann-Whitney *U* test was used for non-normally distributed data, and Student’s *t* test was used for normally distributed data.
