# Supplementary material for: Elevated Serum Leptin Levels as a Predictive Marker for Polycystic Ovary Syndrome
Source: Front Endocrinol (Lausanne). 2022 Mar 9;13:845165. doi: 10.3389/fendo.2022.845165 (PMC8959426; doi:10.3389/fendo.2022.845165)
Supplement: Supplementary file 2 [file Table_2.docx]

**Supplementary Table 2.** Description of the study participants subdivided by different levels of BMI.

|  | Lean | | | Overweight/obese | | |
| --- | --- | --- | --- | --- | --- | --- |
|  | Control (n = 48) | PCOS (n = 15) | *P*-value | Control (n = 91) | PCOS (n = 74) | *P*-value |
| Age (year) | 31.00 (29.00-34.00) | 32.00 (30.00-33.00) | NS | 31.00 (29.00-33.00) | 32.00 (30.00-34.00) | NS |
| BMI (kg/m^2^) | 21.55 (20.70-22.28) | 21.20 (20.20-22.10) | NS | 25.20 (24.00-27.55) | 26.54 (24.68-29.00) | *P* < 0.05 |
| Leptin (ng/mL) | 6.68 (4.28-8.43) | 9.80 (7.94-14.26) | *P* < 0.05 | 13.29 (9.01-16.82) | 15.68 (13.13-19.37) | *P* < 0.001 |
| FPG (mM) | 5.04 (4.89-5.19) | 5.07 (4.77-5.32) | NS | 5.31 (5.06-5.59) | 5.22 (4.90-5.51) | NS |
| FSI (mIU/L) | 8.35 (6.63-10.45) | 9.70 (7.50-12.40) | NS | 13.20 (10.10-16.30) | 14.95 (11.25-22.33) | *P* < 0.05 |
| HOMA-IR | 1.90 (1.46-2.41) | 2.06 (1.66-2.74) | NS | 3.14 (2.33-4.26) | 3.57 (2.60-5.10) | *P* < 0.05 |
| Free testosterone (nmol/L) | 0.019 (0.015-0.024) | 0.031 (0.021-0.039) | *P* < 0.01 | 0.025 (0.019-0.034) | 0.031 (0.022-0.040) | *P* < 0.01 |
| DHEAS (nmol/L) | 2368 (1826-3397) | 5433 (4160-6417) | *P* < 0.001 | 3336 (2422-4502) | 4047 (2920-5835) | *P* < 0.05 |
| Total testosterone (ng/mL) | 0.45 (0.35-0.56) | 0.63 (0.51-0.70) | *P* < 0.001 | 0.51 (0.37-0.61) | 0.70 (0.54-0.85) | *P* < 0.001 |
| FSH (mIU/L) | 7.05 (5.61-8.57) | 6.46 (6.05-7.23) | NS | 7.16 (5.70-8.11) | 6.31 (5.14-7.74) | *P* < 0.05 |
| LH (mIU/L) | 3.84 (2.87-5.02) | 12.57 (5.35-17.48) | *P* < 0.001 | 3.65 (2.96-5.29) | 10.11 (5.67-14.49) | *P* < 0.001 |
| LDL-C (mM) | 2.52 ± 0.72 | 2.90 ± 0.81 | NS | 2.76 ± 0.67 | 3.10 ± 0.72 | *P* < 0.01 |
| HDL-C (mM) | 1.32 (1.20-1.60) | 1.19 (1.04-1.40) | NS | 1.14 (1.00-1.33) | 1.10 (0.94-1.35) | NS |
| Prolactin (ng/mL) | 9.50 (8.14-11.76) | 9.35 (6.26-10.67) | NS | 11.10 (8.24-14.23) | 9.33 (7.32-12.46) | *P* < 0.05 |
| Progestin (mIU/mL) | 0.55 (0.35-0.83) | 0.55 (0.35-0.68) | NS | 0.53 (0.34-0.71) | 0.56 (0.36-1.06) | NS |
| AMH (ng/mL) | 2.53 (1.18-4.18) | 9.15 (6.54-12.40) | *P* < 0.001 | 3.24 (1.88-5.04) | 7.99 (4.86-11.43) | *P* < 0.001 |
| Estrogen (pg/mL) | 51.50 (40.25-68.75) | 51.00 (35.00-97.00) | NS | 45.00 (35.00-59.00) | 53.00 (40.00-67.00) | *P* < 0.05 |
| Total cholesterol (mM) | 4.37 ± 0.85 | 4.70 ± 0.88 | NS | 4.49 ± 0.78 | 4.93 ± 0.84 | *P* < 0.01 |
| Triglycerides (mM) | 0.76 (0.60-1.00) | 1.01 (0.74-1.83) | *P* < 0.05 | 1.17 (0.78-1.88) | 1.54 (1.04-2.04) | *P* < 0.05 |
| TSH (µIU/mL) | 1.93 (1.56-2.50) | 1.87 (1.16-2.35) | NS | 1.75 (1.45-2.71) | 1.72 (1.22-2.24) | NS |

**Abbreviations:** AMH, anti-Müllerian hormone; BMI, body mass index; DHEAS, dehydroepiandrosterone sulfate; FSH, follicle-stimulating hormone; FSI, fasting serum insulin; FPG, fasting plasma glucose; HDL-C, high-density lipoprotein cholesterol; HOMA-IR, homeostasis model assessment of insulin resistance; LDL-C, low-density lipoprotein cholesterol; LH, luteinizing hormone; TSH, thyroid-stimulating hormone; NS, not significant. Mean ± standard deviation or median (interquartile range) are shown. The Mann-Whitney *U* test was used for non-normally distributed data, and Student’s *t* test was used for normally distributed data.
