# Supplementary material for: Elevated Serum Leptin Levels as a Predictive Marker for Polycystic Ovary Syndrome
Source: Front Endocrinol (Lausanne). 2022 Mar 9;13:845165. doi: 10.3389/fendo.2022.845165 (PMC8959426; doi:10.3389/fendo.2022.845165)
Supplement: Supplementary file 3 [file Table_3.docx]

**Supplementary Table 3.** Correlation between leptin levels and clinical and metabolic profiles of patients with PCOS.

| Correlation of  leptin levels and | PCOS (n = 89) | |
| --- | --- | --- |
|  | r | *P*-value |
| Age | -0.203 | 0.169 |
| BMI (kg/m^2^) | 0.695 | 0.001^**^ |
| FPG (mM) | 0.286 | 0.007^**^ |
| FSI (mIU/L) | 0.383 | 0.001^**^ |
| HOMA-IR | 0.415 | 0.001^**^ |
| Free testosterone (nmol/L) | 0.074 | 0.492 |
| DHEAS (nmol/L) | -0.117 | 0.273 |
| Total testosterone (ng/mL) | 0.308 | 0.003^**^ |
| Total cholesterol (mM) | 0.025 | 0.819 |
| Triglycerides (mM) | 0.152 | 0.155 |
| LDL-C (mM) | -0.042 | 0.694 |
| HDL-C (mM) | -0.115 | 0.283 |
| Prolactin (ng/mL) | -0.062 | 0.563 |
| Progestin (mIU/mL) | 0.069 | 0.519 |
| AMH (ng/mL) | -0.144 | 0.180 |
| Estrogen (pg/mL) | 0.015 | 0.894 |
| TSH (µIU/mL) | -0.046 | 0.667 |
| LH (mIU/L) | -0.022 | 0.834 |
| FSH (mIU/L) | -0.082 | 0.446 |

**Abbreviations:** AMH, anti-Müllerian hormone; BMI, body mass index; DHEAS, dehydroepiandrosterone sulfate; FSH, follicle-stimulating hormone; FSI, fasting serum insulin; FPG, fasting plasma glucose; HDL-C, high-density lipoprotein cholesterol; HOMA-IR, homeostasis model assessment of insulin resistance; LDL-C, low-density lipoprotein cholesterol; LH, luteinizing hormone; TSH, thyroid-stimulating hormone; ^*^: *P* < 0.05; ^**^: *P* < 0.01.
